# Extracellular Vesicles from a Biofilm of a Clinical Isolate of *Candida albicans* Negatively Impact on *Klebsiella pneumoniae* Adherence and Biofilm Formation

**DOI:** 10.3390/antibiotics13010080

**Published:** 2024-01-15

**Authors:** Marianna Imparato, Angela Maione, Annalisa Buonanno, Renato Gesuele, Noemi Gallucci, Maria Michela Corsaro, Luigi Paduano, Angela Casillo, Marco Guida, Emilia Galdiero, Elisabetta de Alteriis

**Affiliations:** 1 Department of Biology, University of Naples ‘Federico II’, Via Cinthia, 80126 Naples, Italy; marianna.imparato@unina.it (M.I.); angela.maione@unina.it (A.M.); annalisa.buonanno@unina.it (A.B.); renato.gesuele@unina.it (R.G.); marco.guida@unina.it (M.G.); elisabetta.dealteriis@unina.it (E.d.A.); 2 Department of Chemical Sciences, University of Naples “Federico II”, Via Cintia 4, 80126 Naples, Italy; noemi.gallucci@unina.it (N.G.); corsaro@unina.it (M.M.C.); lpaduano@unina.it (L.P.); angela.casillo@unina.it (A.C.); 3 National Biodiversity Future Center (NBFC), 90133 Palermo, Italy; 4 Center for Studies on Bioinspired Agro-Environmental Technology (BAT Center), 80055 Portici, Italy

**Keywords:** extracellular vesicles, pathogenic fungi, candidiasis, biofilm, Gram-negative bacteria

## Abstract

The opportunistic human fungal pathogen *Candida albicans* produces and releases into the surrounding medium extracellular vesicles (EVs), which are involved in some processes as communication between fungal cells and host–pathogen interactions during infection. Here, we have conducted the isolation of EVs produced by a clinical isolate of *C. albicans* during biofilm formation and proved their effect towards the ability of the Gram-negative bacterial pathogen *Klebsiella pneumoniae* to adhere to HaCaT cells and form a biofilm in vitro. The results represent the first evidence of an antagonistic action of fungal EVs against bacteria.

## 1. Introduction

Extracellular vesicles (EVs) are produced by most prokaryotic and eukaryotic species and released into the surrounding medium as an integral part of their lifecycle [1,2]. EVs are involved in numerous functions such as nutrient acquisition, gene transfer, biofilm formation, toxin release and cell-to-cell communications [3,4,5,6,7].

Both pathogenic and non-pathogenic fungi produce and release of EVs, with a large body of literature particularly focusing on their role in host-pathogen interactions and immunomodulatory response [8,9,10]. Most information is available on EVs from *Candida albicans* [11,12,13], the main fungal infectious agent in humans, and also other non-albicans *Candida* (NAC) species such as *C. glabrata, C. tropicalis, C. parapsilosis* [14], and *C. auris* [15] growing in planktonic cultures, whereas only few studies report EVs occurrence and production in *Candida* biofilms. Biofilms are surface-associated cellular communities encased in a polymeric matrix, representing a common microbial lifestyle, which result in extremely resistant to antimicrobials, so having a major role in recalcitrant infections.

*C. albicans* EV content differs substantially as the fungus switches from the planktonic to biofilm growth mode [16]. EVs derived from biofilms of wild type strain were able to restore the biofilm production and fluconazole tolerance in mutant strains, showing that biofilm-derived EVs are different in size, quantities, and cargo during the biofilm developmental phases. In particular, the presence of a set of proteins critical for key biofilm community functions was reported [16]. In a recent work [17], EVs produced by *C. albicans* were found to inhibit its biofilm formation and the yeast-to-hyphae transition.. The stop of filamentation and the formation of pseudo-hyphae were attributed to some EVs components such as sesquiterpenes, diterpenes, and medium-chain fatty acids [17].

Also, in the case of NAC species, biofilm-derived EVs were reported to show dissimilarities in size and content if compared to EVs from the corresponding planktonic cultures [14,15,18].

From the point of view of applications, EVs may have great potential in the field of infectious diseases to be proposed for the development of EV-based vaccines and antimicrobial entities [19]. The vesicles produced by fungal biofilms can sequester within them the same drugs used to fight fungal infections, so recent studies have proposed that a useful therapeutic target should be the production and release of these vesicles produced by biofilms [20].

Indeed, in mammalian, plant, and microbial hosts EVs are produced in response to a pathogenic attack, functioning as discrete carriers for molecules addressed against enemies and competitors [21]. Since ‘90 many examples of antimicrobial and antibiofilm activities associated with bacterial EVs have been reported and attributed to the presence of different compounds such as bacteriocins [22], hydrolytic enzymes [23], antibiotics [24,25] and small interfering RNAs [26].

In the case of fungi and *C. albicans*, no antagonistic effects of EVs on other species have been explored so far. Instead, in a recent work [17], EVs produced by *C. albicans* were found to inhibit its biofilm formation and the yeast-to-hyphae transition. Also, EVs attenuated *Candida* virulence in *Galleria mellonella*. The stop of filamentation and the formation of pseudo-hyphae were attributed to some EVs components such as sesquiterpenes, diterpenes, and medium-chain fatty acids [11].

Therefore, taking into account the reported composition of *Candida* EVs [11,26], and considering the increasing need for alternative antibacterial strategies, we investigated whether EVs derived from *C. albicans* biofilm could have potential antagonistic activity against a representative bacterial species, such as the opportunistic Gram-negative pathogen *Klebsiella pneumoniae*. Indeed, it has been recognized in the “ESKAPE” (*Enterococcus faecium*, *Staphylococcus aureus*, *Klebsiella pneumoniae*, *Acinetobacter baumannii*, *Pseudomonas aeruginosa*, and *Enterobacter* species), group of bacteria since “escaping” from the action of several antibiotics. *K. pneumoniae* causes nosocomial and community-acquired infections, leading to increasing mortality, especially in immunocompromised patients [27]. *Klebsiella* infections are often associated with the occurrence of biofilms on biotic and abiotic surfaces [28]. In this work, we isolated EVs from the biofilm of a clinical *C. albicans* isolate with the main purpose of exploring the effect of EVs on both the capacity to inhibit the *K. pneumoniae* biofilm production phases and to adhere to human cells in vitro. Also, *Candida* EVs were tested for the prevention of *K. pneumoniae* infection in vivo, by using the *Galleria mellonella* model.

## 2. Results

### 2.1. Extracellular Vesicle Isolation, Visualization and Characterization

**Figure 1:**
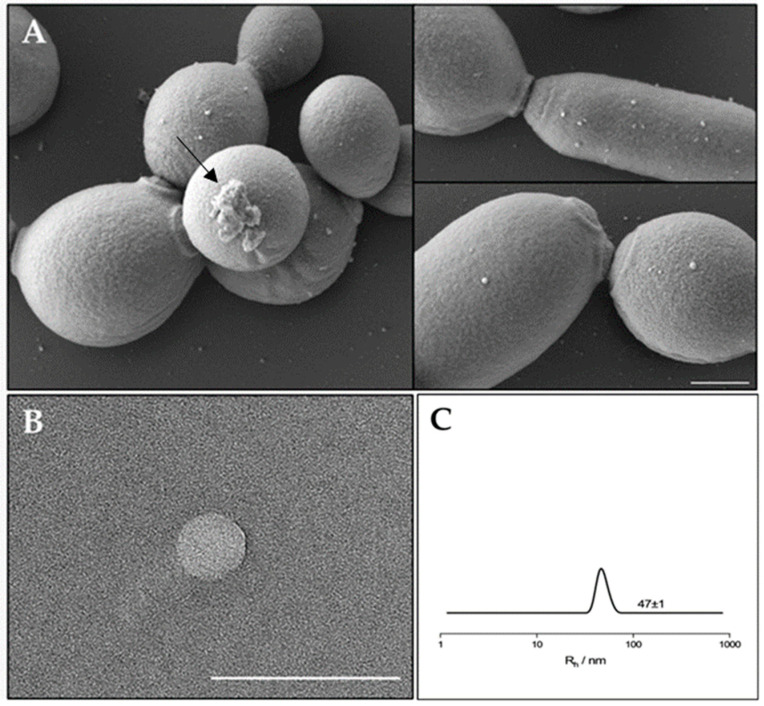
(**A**) SEM visualization of *C. albicans* biofilm showing EVs on the surface. The arrow indicates a residue of extracellular matrix of the biofilm. Bar corresponds to 1 mm. (**B**) TEM photograph of an isolated extracellular vesicle from *C. albicans* biofilm. Bar corresponds to 200 nm. (**C**) Hydrodynamic radius distribution of an EVs suspension in PBS.

In order to evaluate the EVs production and composition, EVs were isolated from a mature biofilm of *C. albicans* clinical isolate grown for 72 h. It was reported that *C. albicans* for biofilm formation produced a preponderance of EVs that increased in number as the biofilm matured and developed an extracellular matrix [16]. Similar results were observed for the *C. albicans* clinical isolate biofilm, which was visualized by SEM. As reported in Figure 1 (panel A) SEM visualization clearly showed extracellular vesicles on the surface of sessile yeast cells and pseudo-hyphae. In Figure 1 (panel B) a transmission electron microscopy image of an isolated vesicle is shown. To better determine the size of the EVs in suspension, Dynamic Light Scattering (DLS) measurements were conducted (Figure 1, panel C). The analysis clearly shows the presence of a single population with a hydrodynamic radius of about 47 nm.

The average amounts of proteins, carbohydrates and phospholipids in our sample of isolated EVs from the *C. albicans* biofilm is reported in Table 1. In the following experiments with EVs, their amount was calculated on the basis of protein content, considering the average protein concentration of 73.4 μg mL^−1^.

**Figure 2:**
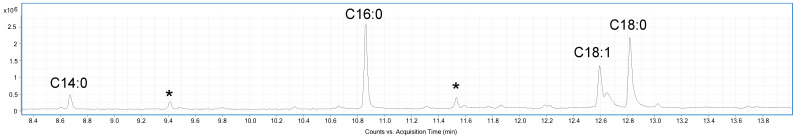
GC-MS chromatogram of FAMEs from *C. albicans* EVs. Peaks marked with an asterisk are contaminants.

A sample of the EVs was analysed by GC-MS (Gas Chromatography-Mass Spectrometry) after derivatization in acetylated methyl glycosides (AMGs) and fatty acid methyl esters (FAMEs) to determine the composition in monosaccharides and fatty acids, respectively. The GC-MS chromatogram reported in Figure 2, suggested the presence of tetradecanoic acid (C14:0), hexadecanoic acid (C16:0), octadecanoic acid (18:0), and octadecenoic acid (C18:1). Furthermore, the analysis of AMGs revealed the presence of traces of glucose, mannose, and galactose, probably attributable to the cell wall polysaccharides, and glycoproteins associated to the surface of EVs [29].

### 2.2. Effect of EVs on K. pneumoniae Biofilm Formation

**Figure 3:**
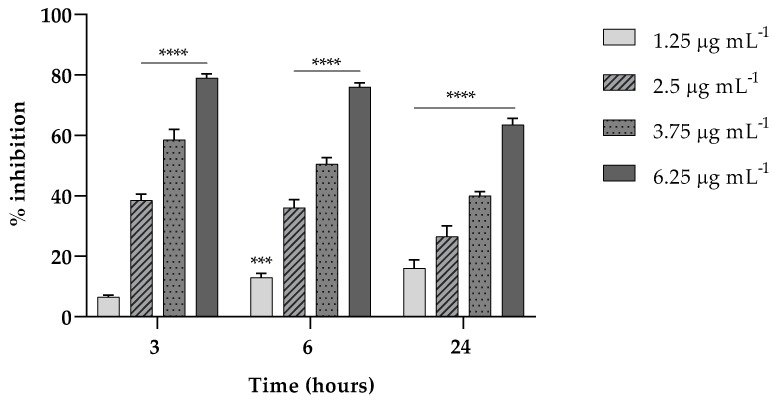
Fungal EVs (1.25; 2.5; 3.75; 6.25 µg mL^−1^, based on protein content) attenuate *K. pneumoniae* biofilm formation at different times of exposition (3, 6 and 24 h). Data were normalized to control and the results are expressed as percentage of inhibition compared to control (*** = *p* < 0.001, **** = *p* < 0.0001, Tukey’s test); Each experiment was replicated three times and error bar represent SD.

Here, we investigated the ability of EVs to interfere in the first two stages (3 and 6 h) of *K. pneumoniae* attachment on a polystyrene surface and in the biofilm formation at 24 h. As reported in Figure 3, in the attachment assay, EVs exerted a percent of inhibition on the adhesion capability of *K. pneumoniae* of about the 80% (*p* < 0.0001) both at 3 and 6 h, at the highest EV concentration tested.

Also, at such concentration, the ability of EVs at the same concentrations was accounted to inhibit the formation of a mature biofilm of about 65% (*p* < 0.0001) reduction compared to the untreated biofilm (Figure 3).

### 2.3. Cytotoxicity of EVs vs. HaCaT Cells

**Figure 4:**
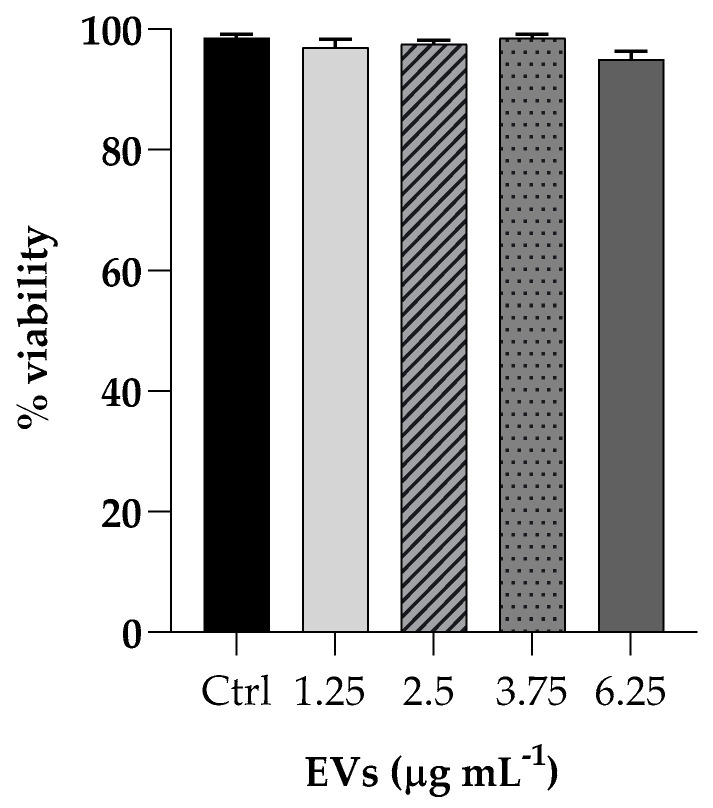
Cell viability of HaCaT cells upon exposure to different concentrations of EVs (1.25; 2.5; 3.75; 6.25 µg mL^−1^). The percentages of cell viability for each of the treatments were calculated in comparison to the control. Each experiment was replicated three times and error bar represent SD.

The effect of EVs was evaluated on cell viability by incubating HaCaT cells (non-tumorigenic human keratinocyte cells) with different EVs concentrations (ranging from 1.25 to 6.25 mg mL^−1^ based on protein content) for 3 h and cytotoxicity activity was analyzed by the MTT assay. Cell viability, expressed as a percentage of viability of non-treated cells, showed no toxicity at all concentrations tested (Figure 4).

### 2.4. C. albicans EVs Prevents K. pneumoniae from Adhering to Human Keratinocyte Cells

**Figure 5:**
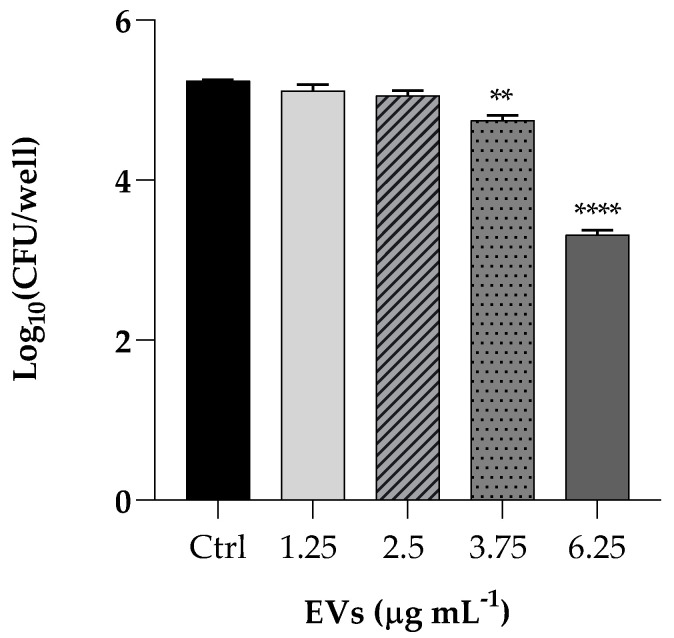
Assessment of anti-adhesion attributes of EVs used at the same concentrations of cytotoxicity assay (1.25; 2.5; 3.75; 6.25 µg mL^−1^). Treatment of HaCaT cells with *K. pneumoniae* only was used as positive control. Data are average of three experiments analyzed in triplicate and error bar represent SD (** = *p* < 0.01, **** = *p* < 0.0001, Tukey’s test).

We performed a *K. pneumoniae* adhesion assay by pretreating HaCaT cells with EVs at concentrations of 1.25, 2.5, 3.75 and 6.25 µg mL^−1^ (based on protein content) Following incubation of the resulting culture, the bacterial cells adhered to the HaCaT cells decreased of 2 Log (*p* < 0.0001) after pretreatment with the highest EVS concentration, resulting in an attenuated adhesion, as shown in Figure 5.

### 2.5. G. mellonella Survival and Infection

After ascertaining the effect of EVs from *C. albicans* on *K. pneumoniae* in vitro, we performed an in vivo assay, evaluating the capacity of the EVs preparation to protect *G. mellonella* larvae from bacterial infection.

**Figure 6:**
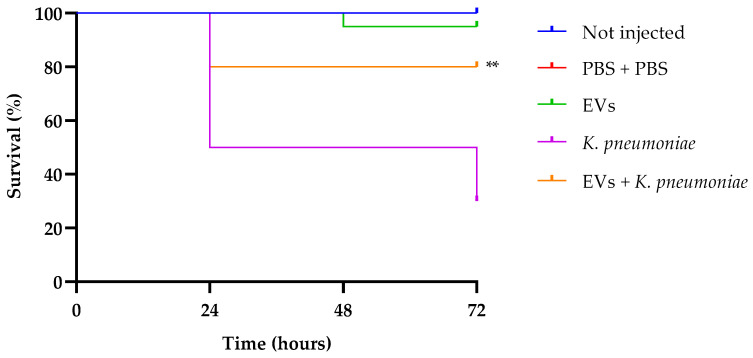
Kaplan–Meier plots of survival curves of *G. mellonella* larvae infected with *K. pneumoniae* (1 × 10^3^ CFU/larva) alone or in the presence of EVs (6.25 µg per larva based on protein content). Survival curves of intact larvae (control) and larvae injected with PBS are also reported. ** Represents significant difference vs. larvae injected with *K. pneumoniae* (*p* < 0.001 Log-rank Mantel-Cox test).

In our previous research, we standardized a *K. pneumoniae* inoculum of 1 × 10^3^ CFU per larva as enough to infect larvae, and monitored animal survival for a 72 h period [30]. Larval survival analysis (Figure 6) indicates that treatment with EVs alone did not show any significant effect compared to the two control groups (intact larvae and larvae injected with PBS alone, presenting about 100% of survival up to 72 h of observation. Instead, infected larvae showed a survival of 30% after the same time. Interestingly, groups that received a pretreatment with EVs and after 2 h were infected with *K. pneumoniae*, showed an increased survival of about 50%, this result confirming the excellent performance of EVs as a prophylactic anti-infectious agent.

## 3. Discussion

Fungal infections are a growing global public health problem, especially for immunocompromised patients with HIV infection and cancer [31]. *Candida albicans* infections including superficial mucosal infections, skin infections, and bloodstream infections are reported to have mortality rates greater than 40% and to be one of the most common coinfected fungal species with SARS-CoV-2 in COVID-19 [32]. In fungi, EVs production carrying a mixture of lipids, nucleic acids, proteins, carbohydrates, toxins and other metabolites EV complex cargo, is tightly regulated and varies depending on the origin of cells and isolation method [33]. EVs production is considered one of the virulence mechanisms of fungal pathogenesis, being the vesicle packaging advantageous compared to a soluble secretion of components directly into the extracellular milieu, because vesicles favor the protection of molecules sensitive to extracellular degradation in the host [8]. It is reported that EVs produced by *C. albicans* activate phagocytosis by macrophages, and such activation was induced by nitric oxide production and balanced by the inflammatory profile measured by cytokines expression [11].

Among the many different functions attributed to EVs, their potential role of defense against competitors has been scarcely investigated and restricted to bacterial EVs [19]. In particular, the potential antagonistic activity of fungal EVs against bacteria has not been explored so far, though fungal-bacterial co-infections are very common and often associated to the formation of polymicrobial biofilms [34]. In this view, EVs could have a putative role of protecting the fungus against bacterial competitors present in the same ecological niche.

Starting from these considerations and given the increasing requirement for alternative antimicrobial/antibiofilm strategies, in this work we investigated the impact of EVs isolated from a biofilm of a clinical isolate of *C. albicans* against *K. pneumoniae*.

First, we visualized EVs on the cell surface of a mature biofilm of *C. albicans* clinical isolate and then we isolated such EVs. The size of EVs was confirmed by DLS measurements.

Our data clearly demonstrate that the EV preparation obtained from a mature *C. albicans* biofilm had a high biocompatibility, being non-toxic for both mammalian cells and insect larvae. EVs were able to affect the formation of a *K. pneumoniae* biofilm in vitro, as well as to inhibit adherence of the bacterium to mammalian cells. It has been previously reported [17] that when administered to *G. mellonella*, *C. albicans* EVs can confer protection in a dose-dependent manner against a subsequent challenge with *C. albicans* yeasts. At present, this study is the first to investigate the antibacterial properties of EVs from a clinical isolate of *Candida* using *G. mellonella*. The anti-*Klebsiella* effect of EVs was evident since the EV preparation protected *G. mellonella* larvae from bacterial infection.

The main components of the EVs from the biofilm of the *Candida* clinical isolate were carbohydrates, proteins and phospholipids, according to the average composition of *Candida* EVs reported by other authors [13,14,16]. More in detail, preliminary analyses evidenced in our sample the occurrence of C14:0, C16:0, C18:1 and C18:0, together with traces of neutral monosaccharides.

Fatty acids are well-known as antimicrobial agents, as well as able to control biofilm formation of different microorganisms [35,36]. Their antibiofilm action depends on the cell type, and may affect cell membrane fluidity, EPS amount, fimbriae or hyphae formation, and cell-to-cell signaling by modulating QS systems [36]. In the specific case of *K. pneumoniae*, caprylic acid (octanoic acid) has been reported to interfere with MrkA and GalF proteins, which are related to biofilm and capsule formation, respectively [37]. Other more recent studies report the effect of prevention of the same acid against the formation of *K. pneumoniae* biofilm [38].

Apart from fatty acids, we cannot exclude the occurrence in our EV preparation of other lipid or peptide moieties, which could contribute to the observed anti-*Klebsiella* effect. A further investigation is necessary to provide a more detailed characterization of the molecular cargo, also aiming at enlarging the spectrum of bacterial targets.

In conclusion, our results represent first evidence of the antagonistic activity of *Candida* EVs, also associated with a high biocompatibility, both relevant properties in the perspective of their potential use as source or delivery systems for antimicrobial/antibiofilm compounds.

## 4. Materials and Methods

### 4.1. Strains, Cells and Culture Conditions

A clinical isolate of *Candida albicans* from our laboratory collection [39] molecularly identified as *Candida albicans* SKH402 (≥98% using nucleotide blast at GenBank https://www.ncbi.nlm.nih.gov/genbank/ accessed on 9 January 2023) was stored in 15% glycerol frozen at −80 °C and routinely maintained on YPD agar plates (1% yeast extract, 2% peptone, 2% dextrose, 2% agar). Liquid planktonic cultures were grown in Tryptic Soy Broth (TSB) supplemented with 1% *w/v* glucose (VWR Chemicals, Radnor, PA, USA) for 18 h at 37 °C on an orbital rotary shaker.

*C. albicans* biofilm was allowed to form on the surface of eight Roux polystyrene flasks, each containing 300 mL of filter-sterilized Roswell Park Memorial Institute medium 1640 (RPMI), buffered with 4-morpholinepropanesulfonic acid (MOPS). Flasks were inoculated with 1 mL (10^9^) of *C. albicans* cells collected from a 18 h pre-culture in RPMI and incubated in static conditions at 37 °C for 72 h. Biofilm mass was evaluated by scraping the surface of the flasks, cells collected and evaluated by total cell count on YPD agar plates.

*Klebsiella pneumoniae* ATCC 10031 was kept on tryptic soy agar plates (TSA, VWR chemicals, Leuven, Belgium). For biofilm formation, colonies were picked up and inoculated into liquid tryptic soy broth (TSB, VWR chemicals, Leuven, Belgium) at 37 °C for 24 h under shaking conditions (200 rpm), then washed twice using sterile phosphate-buffered saline (PBS) and adjusted to 10^6^ cells mL^−1^ in the same culture medium.

HaCAT cells (non-tumorigenic human keratinocyte cells) were obtained from the ATCC (American Type Culture Collection, Manassas, VA, USA). They were maintained in Dulbecco’s Modified Eagle Medium (DMEM, Sigma Aldrich Co., St. Louis, MO, USA), supplemented with 10% Fetal Bovine Serum, 1% L-glutamine and 1% penicillin/streptomycin (Sigma Aldrich) in a humidified incubator at 37 °C and 5% CO_2_. The cells were detached with Trypsin/EDTA solution (Sigma Aldrich) and cultured into new flasks after reaching 70–80% confluency. The medium was changed twice a week.

### 4.2. Extracellular Vesicle Isolation

EVs were recovered and isolated from the supernatants of a 72 h biofilm of *C. albicans*. For EVs isolation the protocol by Karkowska-Kuleta et al. [13] was followed with some modifications. For that, 2400 mL of supernatants from biofilm cultures (corresponded to 2 × 10^11^ cells) were collected and centrifuged at 5000× *g* for 20 min at 4 °C and also again centrifuged at 15,000× *g* (4 °C) to remove cells and smaller debris, respectively. Each time, cells and pellets were discarded. An Amicon Ultra-15 Centrifugal Filter Unit with a 100 kDa cut off (Sartorius, Vivaspin Turbo 15) was used to concentrate the supernatants with the addition of a complete protease inhibitor cocktail (Roche, Sigma-Aldrich). After centrifuged for 5 min at 5000× *g*, the samples were filtered using an Ultrafree-CL Centrifugal Filter with pore size of 0.65 mm (Sigma-Aldrich). To confirm the absence of any remaining fungal cells, the aliquots were spread onto YPD agar plates after this step. Concentrated supernatants were then ultracentrifuged at 4 °C for 1.5 h at 10,000× *g*, using a fixed-angle type 50.2 Ti Rotor and polycarbonate thick wall centrifuge tubes (13 × 64 mm) with 13 mm diameter Delrin tube adapters in an OptimaTM L-90K Ultracentrifuge (all from Beckman Coulter, Brea, CA, USA). The EV pellet obtained was washed with phosphate buffered saline (PBS) buffer and then subjected to another ultracentrifugation step under the same conditions. After discarding the supernatant, the pellet containing EVs was resuspended in 400 μL of PBS, transferred to an Eppendorf tube, and stored at 4 °C for future use.

The presence of EVs was confirmed using dynamic light scattering (DLS).

### 4.3. Visualization of C. albicans Biofilm by SEM

To visualize the 72 h *C. albicans* biofilm, the protocol by Zarnowski et al. [16] was followed, with some modifications. Briefly, 40 μL of an inoculum of 10^8^ Candida cellsmL^−1^ in RPMI–MOPS was added to the 13 mm diameter coverslips in a 12 microwell-plate and incubated for 60 min at 37 °C. Then, 1 mL RPMI–MOPS was added to each well, and the microplate incubated for 72 h, renewing the medium every 24 h. Coverslips were then washed with PBS, fixed overnight with 3% *v/v* glutaraldehyde at 4 °C, then washed with PBS and post-fixed in 1% aqueous solution of osmium for 90 min at room temperature. Then, samples were dehydrated in a series of graded alcohols, dried to the critical drying point, and finally coated with gold. A scanning electron microscope (operating under a high vacuum with 10 kV (QUANTA 200 ESEM FEI Europe Company, Eindhoven, The Netherlands). was used to observe the specimens.

### 4.4. Transmission Electron Microscopy (TEM)

Specimens were prepared for TEM using the conventional negative staining procedure. About 7–10 mL of EV sample were placed onto a formvar-coated grid and allowed to settle for 30 min at room temperature. Then, grids were washed with bi-distilled water, and a drop of diluted uranyl acetate replacement (UAR, Electron Microscopy Science, Hatfield, PA, USA) was applied. After 10 s, the excess was removed with filter paper. The grids were observed with a TECNAIG2 S-twin apparatus (FEI, Thermo Fisher, Waltham, MA, USA) at 120 kV.

### 4.5. Dynamic Light Scattering (DLS)

To confirm the presence of EVs in supernatant and determine their size by DLS. Measurements were performed using a home-made instrument composed of a Photocor compact goniometer (Moscow, Russia), an SMD 6000 Laser Quantum 50 mW light source (Laser Quantum, Heaton Mersey, UK) operating at 532.5 nm, a photomultiplier (PMT-120-OP/B), and a correlator (Flex02-01D) from Correlator.com (2006) [40,41]. The experiments were carried out at the constant temperature of 25.0 ± 0.1 °C, using a thermostatic bath and at the scattering angle θ of 90°.

### 4.6. Proteins, Carbohydrates, and Phospholipids Determinations

Aliquots of the EVs suspension obtained as reported above were used to measure the protein, carbohydrate and phospholipid concentrations. Proteins were determined by the Bio-Rad Protein assay Kit (Bio-Rad, CA, USA). For carbohydrate and phospholipids determinations, the Carbohydrate Assay Kit (MAK104, Sigma-Aldrich) and Phospholipid Assay Kit (MAK122, Sigma-Aldrich) were used, respectively, following the manufacturer’s instructions, and measuring sample absorbance with Synergy H1 Microplate Reader (BioTek Instruments, Winooski, VT, USA).

For GC-MS derivatization, EVs sample was treated with 1 mL of hydrogen chloride—methanol solution 1.25 M and the methanolysis was performed at 80 °C for 16 h. The fatty acids were recovered by three extractions with hexane. The monosaccharides obtained were acetylated with acetic anhydride (Ac_2_O) in pyridine. The derivatives were analyzed by using an Agilent instrument gas chromatograph 6850A equipped with a mass selective detector 5973N and a Zebron ZB-5 capillary column (Phenomenex, Bologna, Italy 30 m × 0.25 mm i.d., flow rate 1 mL/min, He as carrier gas), as already reported [42].

### 4.7. Biofilm Assays

For *K. pneumoniae* biofilm production and quantification a previous protocol was used [43]. Briefly, bacteria cells at concentration of 10^5^ cells mL^−1^ grown in TSB broth were plated into polystyrene 96-well microplates in the presence of different concentrations of EVs (6.25; 2.5 and 1.25 μg mL^−1^ based on protein content) and incubated at 37 °C for 3, 6, and 24 h. After incubation, the non-adhesive cells were eliminated, and the plates were washed three times with PBS. The crystal violet (CV) staining method was used to quantify the residual biofilm biomass [44,45]. In summary, plates were washed three times with PBS, biofilm was fixed at 37 °C for 1 h and finally 200 µL of CV (0.2% *v/v*) was added to well for 15 min. Biofilm was resuspended by adding 300 µL of acetic acid at 32% (*v/v*) and the microtiter plate reader was utilized to measure the absorbance at 570 nm. The percentages of biofilm inhibition were calculated as: % Biofilm reduction = OD_570_ of the control − OD_570_ of the treated)/OD_570_ of the control × 100.

### 4.8. Cytotoxicity Assay

HaCaT cells were seeded in 96-well plates (2 × 10^4^ cells mL^−1^) and incubated for 24 h at 37 °C in 5% CO_2_. The cytotoxicity was evaluated by the 3-(4,5-Dimethylthiazol-2-yl)-2,5-Diphenyltetrazolium Bromide (MTT, Sigma-Aldrich, St. Louis, MO, USA) assay. After one day, cells were treated with different concentrations of EVs (1.25; 2.5; 3.75; 6.25 µg mL^−1^ based on protein content) and incubated for 4 h at 37 °C. After the medium removal, DMSO was used to dissolve the formazan crystals, and the absorbance was measured at 570 nm with a microplate reader (SYNER- GYH4, BioTek, Inc., Winooski, VT, USA). The viability of cells was evaluated compared to the control cells.

### 4.9. Adhesion Assay

The adhesion capacity of *K pneumoniae* to HaCaT cells with and without EVs were evaluated. In brief, 10^6^ cells mL^−1^ of *K. pneumoniae* were seeded into 24-well polystyrene plates and incubated with pre-cultured HaCaT cells in DMEM w/o penicillin/streptomycin for 2 h at 37 °C with or without EVs (1.25; 2.5; 3.75; 6.25 µg mL^−1^ based on protein content). Then, the cells were washed with PBS, and 100 μL of a solution of Trypsin/EDTA (Sigma Aldrich Co., St. Louis, MO, USA) was added to each well to detach cells. After 5 min at 37 °C, 1 mL of DMEM was added and cell suspensions recovered. These were diluted in PBS, plated on TSA and incubated at 37 °C for 24 h to count *K. pneumoniae* cell forming units (CFU). The influence of EVs on the adhesion of *K. pneumoniae* to HaCaT cells was calculated by the number of viable adherent bacteria and expressed by CFU per well.

### 4.10. Galleria Mellonella Survival Assay

Larvae of *G. mellonella* were used to evaluate both the EVs toxicity in vivo and the EVs capacity to protect larvae from *K. pneumoniae* infection. For the survival assay ten groups of 20 randomly chosen larvae with a similar size were selected and injected with 10 μL of the *K. pneumoniae* standardized suspension (1 × 10^3^ cells mL^−1^) or with 10 μL EVs preparation (6.25 μg per larva based on protein content) through inferior left proleg by using a Hamilton syringe. For the prophylactic treatment with EVs, larvae were first treated with EVs and after 2 h of incubation at 37 °C, infected with *K. pneumoniae* via the last right proleg. Sterile PBS alone or intact larvae were used as controls. The injected larvae were kept at 37 °C, and the number of dead subjects per group was monitored daily until 3 days.

### 4.11. Statistical Analyses

GraphPad Prism Software (version 8.02 for Windows, GraphPad Software, La Jolla, CA, USA, www.graphpad.com, accessed on 20 November 2023) was used for statistical analyses. The result was obtained from three independent experiments, which are showed as mean ± standard deviation (SD). The Kaplan–Meier method was used to plot survival curves and by Log-rank (Mantel-Cox) test for comparation between the groups. One or two-way ANOVA following Tukey’s test was used to compare the results. Asterisks show significant differences, (* = *p* < 0.05, ** = *p* < 0.01, *** = *p* < 0.001, **** = *p* < 0.0001).

## Figures and Tables

**Table 1:** Composition of EVs isolated from 72 h *C. albicans* biofilm *.

Proteins (μg)	Carbohydrates (μg Glucose Equiv)	Phospholipids (Nanomoles Lecithin Equiv)
29.3 ± 0.396	26.4 ± 0.495	58.4 ± 0.354

* EVs isolated from 2 × 10 ^11^ cells and suspended in 400 μL.

## Data Availability

Data are contained within the article.
